# The Genetics of Alcohol Metabolism: Role of Alcohol Dehydrogenase and Aldehyde Dehydrogenase Variants

**Published:** 2007

**Authors:** Howard J. Edenberg

**Keywords:** Alcohol and other drug (AOD) use (AODU), abuse and dependence, alcoholism, genetics and heredity, genetic theory of AODU, ethnic group, protective factors, ethanol metabolism, liver, alcohol dehydrogenase (ADH), aldehyde dehydrogenase (ALDH), risk factors, protective factors, alcohol flush reaction

## Abstract

The primary enzymes involved in alcohol metabolism are alcohol dehydrogenase (ADH) and aldehyde dehydrogenase (ALDH). Both enzymes occur in several forms that are encoded by different genes; moreover, there are variants (i.e., alleles) of some of these genes that encode enzymes with different characteristics and which have different ethnic distributions. Which *ADH* or *ALDH* alleles a person carries influence his or her level of alcohol consumption and risk of alcoholism. Researchers to date primarily have studied coding variants in the *ADH1B, ADH1C,* and *ALDH2* genes that are associated with altered kinetic properties of the resulting enzymes. For example, certain *ADH1B* and *ADH1C* alleles encode particularly active ADH enzymes, resulting in more rapid conversion of alcohol (i.e., ethanol) to acetaldehyde; these alleles have a protective effect on the risk of alcoholism. A variant of the ALDH2 gene encodes an essentially inactive ALDH enzyme, resulting in acetaldehyde accumulation and a protective effect. It is becoming clear that noncoding variants in both *ADH* and *ALDH* genes also may influence alcohol metabolism and, consequently, alcoholism risk; the specific nature and effects of these variants still need further study.

The effects of ingested beverage alcohol (i.e., ethanol) on different organs, including the brain, depend on the ethanol concentration achieved and the duration of exposure. Both of these variables, in turn, are affected by the absorption of ethanol into the blood stream and tissues as well as by ethanol metabolism (Hurley et al. 2002). The main site of ethanol metabolism is the liver, although some metabolism also occurs in other tissues and can cause local damage there. The main pathway of ethanol metabolism involves its conversion (i.e., oxidation) to acetaldehyde, a reaction that is mediated (i.e., catalyzed) by enzymes known as alcohol dehydrogenases (ADHs). In a second reaction catalyzed by aldehyde dehydrogenase (ALDH) enzymes, acetaldehyde is oxidized to acetate. Other enzymes, such as cytochrome P450 (e.g., CYP2E1), metabolize a small fraction of the ingested ethanol.

There are multiple ADH and ALDH enzymes that are encoded by different genes (Tables 1 and 3). Some of these genes occur in several variants (i.e., alleles^1^), and the enzymes encoded by these alleles can differ in the rate at which they metabolize ethanol (Table 2) or acetaldehyde or in the levels at which they are produced. These variants have been shown to influence a person’s drinking levels and, consequently, the risk of developing alcohol abuse or dependence (Hurley et al. 2002). Studies have shown that people carrying certain *ADH* and *ALDH* alleles are at significantly reduced risk of becoming alcohol dependent. In fact, these associations are the strongest and most widely reproduced associations of any gene with the risk of alcoholism. As will be discussed later in this article, the alleles encoding the different ADH and ALDH variants are unevenly distributed among ethnic groups.

The mechanism through which ADH and ALDH variants influence alcoholism risk is thought to involve at least local elevation of acetaldehyde levels, resulting either from a more rapid ethanol oxidation (in cases of more active ADH variants) or from slower acetaldehyde oxidation (in cases of less active ALDH variants). Acetaldehyde is a toxic substance whose accumulation leads to a highly aversive reaction that includes facial flushing, nausea, and rapid heart beat (i.e., tachycardia). This reaction is similar to that experienced by alcoholics who consume alcohol after taking disulfiram (Antabuse^®^), a medication that discourages further drinking.

Most of the numerous variants of the *ADH* and *ALDH* genes involve changes of single DNA building blocks (i.e., nucleotides) and therefore are known as single-nucleotide polymorphisms (SNPs). (For sources of data on SNPs, see the Textbox.) Some SNPs occur in those parts of a gene that actually encode the corresponding protein. These SNPs are called coding variations and in many cases result in the generation of enzymes with altered activity. Other SNPs, however, occur outside the coding regions of a gene (i.e., are noncoding variations). Several noncoding variations have been demonstrated to affect the expression of the genes (e.g., Chen et al. 2005; Edenberg et al. 1999), and it is likely that others have the same effect. (For more information on the typical structure of genes and gene expression in higher organisms, see the Sidebar.) Comprehensive analyses of the *ADH* genes recently demonstrated that both coding and noncoding variations in those genes are associated with the risk for alcoholism in European-American families (Edenberg et al. 2006). Therefore, earlier studies of *ADH* and *ALDH* genes and the associated risk of alcoholism should be reexamined in light of the many coding and noncoding variations that have since been identified.

**Sources of Data on Genetic Variations**

There are several excellent sources of data on single-nucleotide polymorphisms (SNPs) and other genetic variations in different populations, including the following:

- dbSNP (www.ncbi.nlm.nih.gov/SNP)
- HapMap (www.hapmap.org)
- ALFRED (alfred.med.yale.edu/Alfred)

This article summarizes current knowledge regarding coding and noncoding variations in the various *ADH* and *ALDH* genes and the possible association of these variations with risk for alcoholism. The article also briefly touches on the differential ethnic distribution of some of these variants. (For more detailed information, readers are referred to the following articles by Ehlers, Scott and Taylor, Moore and colleagues, and Eng and colleagues, which focus on specific ethnic groups.)

## *ADH* Genes and Their Polymorphisms

**Figure 1:**
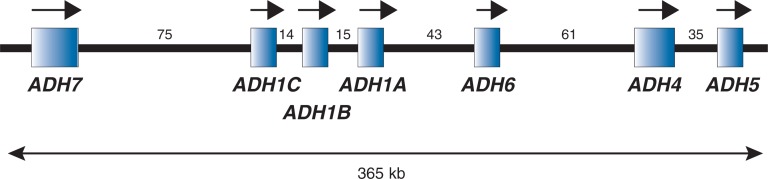
Relative sizes and positions of the seven human alcohol dehydrogenase (*ADH*) genes on the long arm of chromosome 4 (i.e., chromosome 4q). They are shown in the direction in which the genes are transcribed (arrows), but this is opposite to their orientation on chromosome 4q (i.e., *ADH5* is closest to the region where the chromosome arms are joined [i.e., the centromere]). The distances between the genes are indicated in kilobasepairs (kb).

Humans have seven different genes, called *ADH1A*, *ADH1B*, *ADH1C*, *ADH4*, *ADH5*, *ADH6*, and *ADH7,* that encode medium-chain ADHs (see Table 1).^2^ These genes all are aligned along a small region of chromosome 4 (Figure 1). The ADH enzymes they encode function as dimers—that is, the active forms are composed of two subunits. Based on similarities in their amino acid sequences and kinetic properties (e.g., the rate at which ethanol is oxidized), the seven ADH types have been divided into five classes (see Table 1). The three class I genes, *ADH1A*, *ADH1B*, and *ADH1C*, are very closely related; they encode the α, β, and γ subunits, which can form homodimers or heterodimers^3^ that account for most of the ethanol-oxidizing capacity in the liver (Hurley et al. 2002; Lee et al. 2006). *ADH4* encodes π-ADH, which contributes significantly to ethanol oxidation at higher concentrations (Table 2). *ADH5* encodes χ-ADH, a ubiquitously expressed formaldehyde dehydrogenase with very low affinity for ethanol. *ADH6* mRNA is present in fetal and adult liver, but the enzyme has not been isolated from tissue and little is known about it. *ADH7* encodes σ-ADH, which contributes to both ethanol and retinol oxidation.

Researchers have identified SNPs in the *ADH1B* and *ADH1C* genes that result in the production of enzymes with different kinetic properties. These SNPs and their effects have been widely studied in different populations (see other articles in this issue). There are three different *ADH1B* alleles that alter the amino acid sequence of the encoded β subunit (Table 2). The ADH1B*1 allele encodes the β1 subunit that has argi-nine (Arg) at positions 48 and 370;^4^ this is the reference allele. ADH1B*2 encodes the β2 subunit that has the amino acid histidine (His) at position 48; this allele is common in Asians. ADH1B*3 encodes the β3 subunit that has cysteine (Cys) at position 370; this allele primarily is found in people of African descent. In both the β2 and β3 subunits, the amino acid substitutions occur at an amino acid that makes contact with the coenzyme nicotinamide adenine dinucleotide (NAD^+^), which is required for ethanol oxidation (Hurley et al. 2002). In both cases, the substitutions result in enzymes that have a 70- to 80-fold higher turnover rate than the β1 subunit because the coenzyme is released more rapidly at the end of the reaction.

There also are three alleles of the *ADH1C* gene (Table 2). ADH1C*1 encodes the γ1 subunit that has Arg at position 272 and isoleucine (Ile) at position 350. ADH1C*2 encodes the γ2 subunit that shows two differences compared with ADH1C*1—it codes for a glutamine (Gln) at position 272 and a valine (Val) at position 350. In almost all cases, these two SNPs occur together (i.e., are in very high linkage disequilibrium [LD]). ADH consisting of two γ1 subunits (i.e., the γ1γ1 homodimeric enzyme) has a turnover rate that is about 70 percent higher than that of the γ2γ2 enzyme. ADH1C*Thr352 encodes a subunit with threonine at position 352; it has been described in Native Americans (Osier et al. 2002*a*). However, the protein has not been studied.

**Gene Structure and Gene Expression in Higher Organisms**

In higher organisms, including humans, the genes encoding the various components of the body are not just simple stretches of DNA that serve as a template from which proteins are generated. Instead, they have a complex structure involving, in some cases, dozens of pieces of coding sequences interspersed with noncoding sequences. The coding sequences, which are those parts of the gene that actually serve as templates for protein production, are called exons. The intervening noncoding sequences are known as introns (see Figure). In addition to the exons and introns, genes contain regulatory sequences that determine in which cell, at what time, and in what amount the gene is actively converted into the corresponding protein (i.e., is expressed). Most of these regulatory sequences are located in front of (i.e., upstream from) the start of the coding sequences; however, other regulatory elements may be located in introns or even behind (i.e., downstream from) the coding sequences. The promoter is a set of regulatory elements located closely near the start of the gene that also specify the exact start site where conversion of the DNA template into intermediary molecules begins. However, other regulatory elements may be located quite a distance away upstream of the gene.

Converting the genetic information contained in a gene on the DNA into a finished protein product is a complex process involving several steps. The first step is known as transcription. During transcription, certain enzymes “read” the DNA (beginning at a specific start signal and continuing to a specific stop signal) and produce a copy of this DNA segment, which consists of similar building blocks as the DNA and is known as messenger RNA, or mRNA. The mRNA that is produced this way still contains copies of both exons and introns. Next, the introns are removed in a process known as splicing. The spliced mRNA then serves as a template that tells other cell components which protein building blocks (i.e., amino acids) they must link together to form a protein, in a process known as translation. The protein that is generated still does not correspond to the final desired protein but has to undergo posttranslational modifications. For example, all translation products begin with the same amino acid that, in most cases, must be removed from the amino acid chain to obtain the final protein product. Furthermore, many proteins require additional modifications (e.g., the addition of sugar molecules or other chemical groups) in order to become fully functional. The entire process of transcription, splicing, translation, and posttranslational modification is collectively referred to as gene expression. Each step in this process is subject to various regulatory processes.

**Figure:**
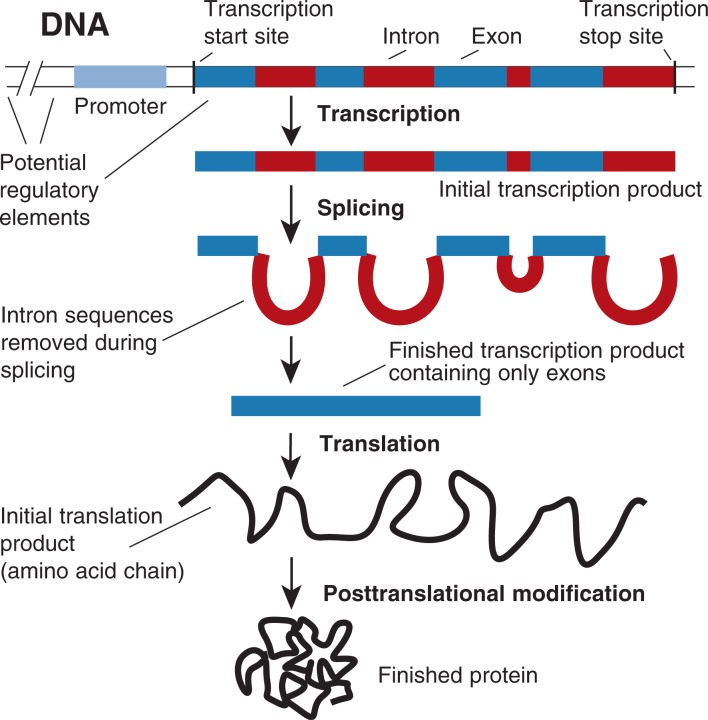

—*Susanne Hiller-Sturmhöfel*

### Effects of Different ADH Variants on Ethanol Metabolism

The differences in the amino acid sequences encoded by the various *ADH1B* and *ADH1C* alleles lead to differences in the predicted rates of ethanol metabolism in the liver. Based on the kinetic properties of the different variants and the estimated ADH enzyme content in liver, researchers have calculated the contribution of the different ADH enzymes to the liver’s ability to oxidize ethanol. These calculations typically are based on a 70-kg man who has a blood alcohol concentration (BAC) of approximately 100 mg/100 mL (0.1 percent, corresponding to approximately 22 mM ethanol, a level that is legally considered intoxicating). If the man carries two copies each of the “reference” ADH1B*1 and ADH1C*1 alleles^5^ (i.e., is homozygous for ADH1B*1 and ADH1C*1), the class I enzymes (that is, homo- and heterodimers of α-, β-, and γ-ADH) together account for approximately 70 percent of the liver’s total ethanol-oxidizing capacity; in addition, π-ADH accounts for nearly 30 percent (Hurley et al. 2002). The ethanol-oxidizing capacities of men with the same weight and BAC, but with different *ADH1B* and *ADH1C* alleles, also have been estimated (Lee et al. 2006). For a man homozygous for the ADH1B*1 and ADH1C*2 alleles, the total ethanol-oxidizing capacity is about 80 percent of the man homozygous for the reference alleles. For a man homozygous for the ADH1B*2 and ADH1C*1 alleles, the oxidizing capacity is almost eight times higher than that of the man homozygous for the reference alleles. For a man homozygous for the ADH1B*3 and ADH1C*1 alleles, the oxidizing capacity is almost twice that of the man homozygous for the reference alleles.

Thus, the presence of the ADH1C*2 allele would be associated with a slightly reduced oxidizing capacity, whereas the presence of ADH1B*2 and ADH1B*3 alleles would be associated with a substantially higher oxidative capacity (i.e., more rapid ethanol oxidation to acetaldehyde). These calculations are rough approximations, however, because they assume that the different alleles are expressed at equal levels. In reality, other factors, including liver size and differences in gene expression, can lead to differences even between individuals carrying the same alleles (i.e., with the same genotype). For example, among healthy young European Americans, almost all of whom are homozygous for ADH1B*1, actual ethanol elimination rates have been shown to differ about four-fold (O’Connor et al. 1998).

### Ethnic Distribution and Protective Effects of ADH Alleles

The ADH1B*2 allele, which is associated with particularly rapid ethanol oxidation, has shown protective effects against alcohol dependence in a variety of populations. In East Asians, in whom the ADH1B*2 allele is found at high frequency, it is protective against alcoholism (Chen et al. 1999*a*; Crabb et al. 2004; Hurley et al. 2002; Luczak et al. 2006; Thomasson et al. 1991; Whitfield 2002). For example, among Chinese living in Taiwan, the odds ratio for developing alcoholism for a person carrying a single ADH1B*2 allele is 0.19, and for a person carrying two ADH1B*2 alleles, 0.12, compared with a person carrying two ADH1B*1 alleles. (This calculation assumes that all subjects are homozygous for the ALDH2*1 allele.) In European or African populations, the ADH1B*2 allele is not very common but also provides protection against alcoholism (Whitfield 2002). Among people of Jewish descent, the ADH1B*2 allele is found at moderate frequencies and reduces binge drinking (Luczak et al. 2002) and risk for alcoholism (Hasin et al. 2002). Overall, the protective effect of ADH1B*2 appears to be weaker in European than in Asian populations (Whitfield 2002). This difference could result from different social and environmental factors. Alternatively, the allele could be co-inherited with other, as-yet-unidentified *ADH* variations that might affect the risk of alcoholism and which could differ between Europeans and Asians.

The ADH1B*3 allele had a significant protective effect on risk for alcoholism in a set of African-American families selected for having multiple alcoholic members (Edenberg et al. 2006). However, the allele was not found among similar European-American families. The ADH1B*3 allele also had a protective effect among Southwest California Indians (Wall et al. 2003). Finally, the ADH1B*3 allele is associated with protection against fetal alcohol syndrome (Hurley et al. 2002; Warren and Li 2005).

The ADH1C*1 allele also appears to have protective effects against alcoholism in Asian populations; however, this protection can be attributed to the fact that this allele usually is co-inherited with the protective ADH1B*2 allele and is not an independent effect of the ADH1C*1 allele (Chen et al. 1999*a*; Choi et al. 2005; Osier et al. 1999).

### Other Variations in *ADH* Genes

The variations in the coding regions of the *ADH* genes discussed above account for only a small fraction of the total variability. Researchers have identified approximately 240 SNPs in the region containing the seven *ADH* genes, most of them in noncoding sequences (i.e., introns) within the genes and in regions flanking the genes. As described later in this section, some of these SNPs affect the level of gene expression.

**Figure 2:**
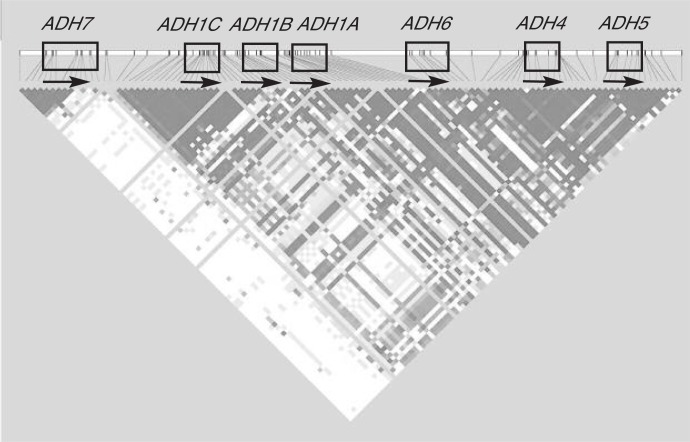
Linkage disequilibrium (LD) among single-nucleotide polymorphisms (SNPs) in the alcohol dehydrogenase (*ADH*) genes. The positions of the genes are indicated at the top. SNPs in which particular combinations of alleles are commonly inherited together have a high LD, depicted in the figure as darker-shaded boxes at the intersections of the SNPs that are being compared. SNPs in which combinations of alleles essentially are random have a lower LD (indicated by lighter shades). SNPs within genes generally are in high LD with each other, whereas SNPs in different genes typically have a lower LD between them. One region of moderately high LD spans most of the genes except *ADH7*. At one site of the *ADH7* gene, frequent rearrangement of the genetic information (i.e., recombination) has occurred so that SNPs upstream of that site are randomly associated with SNPs downstream of that site (as indicated by the area of mostly white boxes). SOURCE: Modified from Edenberg et al. 2006.

SNPs occur as a result of mutations during the course of evolution, so each is initially associated with a particular pattern of other SNPs on the chromosome in which it arose. Over many generations, recombination reshuffles the pattern of SNPs, with closer SNPs less likely to be separated. Therefore, SNPs are not randomly sorted along chromosomes; the nonrandom coin-heritance of alleles, which is called LD, tends to be higher among nearby SNPs. There is strong LD between variants in the *ADH1B* and *ADH1C* genes, which complicates analyses of the effects of individual coding variations in those genes (Chen et al. 1999*a*; Osier et al. 1999, 2002*b*, 2004). The most extensive analysis to date of variations in the *ADH* gene region showed strong LD across nearly the entire region (Figure 2) (Edenberg et al. 2006). The LD was particularly striking in the regions encompassing the *ADH1C*–*ADH1B* and *ADH4*–*ADH5* genes. Conversely, a site of frequent recombination occurs within the *ADH7* gene, which means that variations near the start (i.e., in the 5′ portion) of that gene are nearly randomly associated with variations in the other *ADH* genes (Han et al. 2005; Edenberg et al. 2006). The strong LD over most of the *ADH* gene region means that the coding variations of those genes that have been most frequently studied are probably closely associated with regulatory variations, making it difficult to determine the exact contributions of individual variants—a fact that has not yet been widely appreciated.

With the exception of *ADH7*, all *ADH* genes are expressed in adult liver (for a review, see Edenberg 2000). The level of gene expression, however, is strongly influenced by DNA sequences located directly in front (i.e., upstream) of the transcribed DNA region, as well as in introns within the gene and regions downstream of the gene. For all *ADH* genes, researchers have identified regulatory sequence elements (i.e., promoters) near the transcription start sites (Edenberg 2000). DNA regions that are farther upstream from the genes and which also may contain regulatory elements still need to be examined. Recently, some studies have focused on determining whether different combinations of SNPs (i.e., different haplotypes) in the promoter regions have different effects on gene expression. A single SNP located 136 basepairs (bp) upstream of the translational start site of *ADH4* results in a two-fold difference in gene expression in vitro (Edenberg et al. 1999). For the *ADH1C* gene, expression differs by 40 percent between various haplotypes in a regulatory element located from 3,910 to 3,496 bp upstream of the transcription start site; in addition, a particular combination of three SNPs and a 66-bp insertion/deletion in a region located from 3,496 to 3,008 bp upstream of the gene is associated with a two-fold difference in gene expression (Chen et al. 2005). The effects of other noncoding polymorphisms on gene expression still need to be studied.

Noncoding variations in *ADH* genes affect the risk for alcoholism. A study of 110 SNPs across all seven *ADH* genes demonstrated that variations in *ADH4* most strongly affected risk for alcoholism in European-American families (Edenberg et al. 2006). The variations extended across the entire gene, with the strongest evidence coming from SNPs located between the intron closest to the start of the gene and a region nearly 20,000 bp beyond the end of the *ADH4* gene. Independent studies (Guindalini et al. 2005; Luo et al. 2005, 2006) have confirmed this finding. Non-coding SNPs in the *ADH1A* and *ADH1B* genes also appear to be associated with alcoholism risk in the European-American family sample (Edenberg et al. 2006). Variants in *ADH1C* have been associated with risk in Native Americans (Mulligan et al. 2003). Variations in the *ADH7* gene may affect the risk for alcoholism through interactions with other variants (Osier et al. 2004). Finally, a recent study (Birley et al. 2005) indicated that there is an important site in the region containing the *ADH* genes that affects alcohol metabolism but is not related to the coding variations in *ADH1B* or *ADH1C*.

In summary, there are numerous coding and noncoding variations in the *ADH* genes, at least some of which can affect risk for alcoholism. Additional studies of the full range of variations in these genes will provide a better understanding of the specific effects of individual variations and their impact on the risk for alcoholism.

## *ALDH* Genes and Their Polymorphisms

Two main ALDH enzymes metabolize the acetaldehyde produced during ethanol oxidation (see Table 3) (Crabb et al. 2004; Hurley et al. 2002): ALDH1, which is found in the fluid filling the cells (i.e., the cytosol) and is encoded by the *ALDH1A1* gene, and ALDH2, which is found in the mitochondria and is encoded by the *ALDH2* gene. The *ALDH1A1* gene extends over about 52 kb on chromosome 9, and *ALDH2* extends over 43 kb on chromosome 12. Both genes have a similar structure with 13 exons. Moreover, the proteins they encode are 70 percent identical in sequence and very similar in structure (Hurley et al. 2002).

### Variations in the *ALDH2* Gene

Probably the best known variation of alcohol-metabolizing enzymes is associated with the *ALDH2* gene. A coding variant known as the ALDH2*2 allele leads to the substitution of lysine for glutamate at position 504.^6^ This substitution results in the production of a nearly inactive ALDH2 enzyme that no longer oxidizes acetaldehyde to acetate. Studies on liver extracts demonstrated that the ALDH2*2 variant was nearly dominant—that is, people who carry one ALDH2*1 and one ALDH2*2 allele (i.e., who are heterozygous) have almost no detectable ALDH2 activity in the liver; people who carry two copies of the ALDH2*2 allele (i.e., who are homozygous) have no detectable activity (Crabb et al. 1989). Studies in which the alleles were introduced into cultured cells confirmed this observation (Xiao et al. 1995, 1996). The inactive ALDH2*2 allele is relatively common in people of Chinese, Japanese, and Korean descent but is essentially absent in people of European or African descent (Oota et al. 2004; Hurley et al. 2002). People carrying an ALDH2*2 allele show an alcohol flush reaction, even when they consume only relatively small amounts of alcohol (Peng et al. 2002). In these people, acetaldehyde levels in the blood increase from nearly undetectable levels to levels high enough to trigger the highly aversive reaction that includes severe flushing, nausea, and tachycardia, a reaction similar to that when disulfiram is present.

The presence of even a single ALDH2*2 allele is strongly protective against alcohol dependence. In fact, the protective effect of ALDH2*2 is the most widely reproduced association of a specific gene with alcoholism (Chen et al. 1999*a*; Hurley et al. 2002; Luczak et al. 2006; Thomasson et al. 1991). Compared with a Chinese man carrying two active ALDH2*1 alleles and the two copies of the normal ADH1B*1 allele, the odds ratio for risk for alcoholism for a Chinese man carrying one inactive ALDH2*2 allele and two ADH1B*1 alleles is 0.33. If, in addition to the ALDH2*2 allele, the man also carries at least one overactive ADH1B*2 allele, the odds ratio declines even further, to 0.05 (Chen et al. 1999*a*). In people homozygous for ALDH2*2, the effects of small amounts of alcohol are even more severe (Chen et al. 1999*b*; Peng et al. 1999), and there are almost no documented cases of such people being diagnosed as alcoholic (Chen et al. 1999*b*; Luczak et al. 2006). The severe effects of the ALDH2*2 variant on acetaldehyde levels and alcoholism risk demonstrate that the mitochondrial ALDH2 enzyme normally is the most important enzyme for eliminating acetaldehyde from the body and keeping acetaldehyde levels extremely low.

As with any gene that affects a person’s risk of developing a complex disease, however, the protective effect of the ALDH2*2 allele can be modulated by the environment. This was clearly demonstrated by Higuchi and colleagues (1994). These investigators found that between 1979 and 1992, the fraction of Japanese alcoholics carrying the ALDH2*2 allele increased from 2.5 to 13 percent, indicating that the allele’s protective effect declined over time. In this case, the cause of this reduced protection presumably is a sociological change toward more alcohol consumption in Japan.

### Noncoding *ALDH2* Variants

As with the *ADH* genes, there are many non-coding variations in the *ALDH2* gene. Some of these noncoding variations may affect gene expression, thereby modulating acetaldehyde elimination. For example, one variant in the *ALDH2* promoter region that occurs in all major ethnic groups affects gene expression and may influence the risk for alcoholism (Chou et al. 1999; Harada et al. 1999). Noncoding *ALDH2* variations also could affect ethanol metabolism in people of European or African descent, who rarely carry the ALDH2*2 allele. In fact, a recent study of six SNPs in a European population confirmed the absence of ALDH2*2 but demonstrated that two individual SNPs and a haplotype consisting of the minor alleles at five DNA sites are associated with elevated BACs after oral ingestion of alcohol (Dickson et al. 2006). The SNPs had a small effect on self-reported intoxication but no significant effect on alcohol dependence. Similar studies in other populations clearly are needed to further explore these issues.

### Variations in the *ALDH1A1* Gene

The cytosolic ALDH1 enzyme also contributes to the elimination of acetaldehyde, helping to control acetaldehyde levels even in people with the ALDH2*2 allele. Several promoter polymorphisms in the *ALDH1A1* gene affect gene expression in vitro (Spence et al. 2003). However, these alleles only occur at low frequencies, and their impact on alcoholism risk still is controversial. One study in Southwest California Indians found that people carrying an ALDH1A1*2 allele had lower rates of alcohol dependence and lower maximum number of drinks ever consumed in a 24-hour period (Ehlers et al. 2004). In contrast, in an Australian community-based sample, ALDH1 enzyme activity in blood cells was not associated with alcoholism or the reaction to alcohol (Hansell et al. 2005). This discrepancy in findings could result from the fact that promoters and variations within them act differently in different cell types and that activity levels in blood cells therefore may not reflect activity levels in the liver.

## Summary

*ADH1B* and *ALDH2* are the genes most strongly associated with risk for alcoholism. Coding variants in both of these genes are strongly protective. They probably decrease alcoholism risk by increasing local acetaldehyde levels, either because ethanol is oxidized more rapidly or because acetaldehyde is oxidized more slowly. The exact balance between the rates of ethanol and acetaldehyde oxidation could be crucial in determining acetaldehyde concentrations within cells, such that small differences in the relative activities of ADH and ALDH might produce significant differences in acetaldehyde concentration (Kitson 1999). Because of this delicate balance, the effects of variations in *ADH* and *ALDH* genes on risk for alcoholism can be demonstrated independently—that is, researchers can determine differences in risk between people carrying different alleles of one gene but identical alleles of the other genes (e.g., Chen et al. 1999*b*; Thomasson et al. 1991).

The distribution of *ADH1B* and *ALDH2* coding variants differs greatly among different populations; for both genes, the protective alleles most commonly are found in people of East Asian origin (for more information, see the article by Eng et al. in this issue). Variations in genes encoding other ADH enzymes influence alcoholism risk in other populations. For example, *ADH4* variants strongly affect alcoholism risk in populations of European descent (Edenberg et al. 2006). Furthermore, noncoding variations in various alcohol-metabolizing enzymes likely also affect risk for alcoholism (Edenberg et al. 2006). These noncoding variants should be studied in more populations.

Although variations in individual *ADH* and *ALDH* genes can affect risk for alcoholism, it is important to remember that no one gene determines this risk. An increasing number of genes not related to ethanol metabolism also affect risk (Edenberg and Foroud 2006). Moreover, the contribution of any gene(s) to risk is modulated by other genes as well as by social and environmental factors.

## Figures and Tables

**Table 1:** Alcohol Dehydrogenase (ADH) Genes and Proteins

Official Gene Name^*^	**Old Name^†^**	**Nonstandard Name^‡^**	**Sequence^§^**	**Protein**	**Class^¶^**
*ADH1A*	*ADH1*	*ADH1A*	NM_000667	α	I
*ADH1B*	*ADH2*	*ADH1B*	NM_000668	β	I
*ADH1C*	*ADH3*	*ADH1C*	NM_000669	γ	I
*ADH4*	*ADH4*	*ADH2*	NM_000670	π	II
*ADH5*	*ADH5*	*ADH3*	NM_000671	χ	III
*ADH6*	*ADH6*	*ADH5*	NM_000672	ADH6	V
*ADH7*	*ADH7*	*ADH4*	NM_000673	σ	IV

* Gene symbol approved by the Human Genome Organization (HUGO) Gene Nomenclature Committee (www.gene.ucl.ac.uk/nomenclature/), as used by the National Center for Biotechnology Information (NCBI).

† Original nomenclature.

‡ A set of nonstandard gene names proposed by Duester and colleagues (1999).

§ Reference sequence number as listed in the NCBI RefSeq database (www.ncbi.nlm.nih.gov/RefSeq/).

¶ ADH proteins have been divided into five classes based on sequence and structural similarities.

SOURCE: Modified from Hurley et al. 2002.

**Table 2:** Kinetic Properties of Alcohol Dehydrogenase (ADH) Proteins

Official Gene Name*	**Amino Acid Differences Between Alleles**	**Protein Name**	***K*_m_(ethanol) mM**	**Turnover (min^−1^)**
*ADH1A*		α	4.0	30
ADH1B*1	Arg48, Arg370	β_1_	0.05	4
ADH1B*2	His48, Arg370	β_2_	0.9	350
ADH1B*3	Arg48, Cys370	β_3_	40	300
ADH1C*1	Arg272, Ile350	γ_1_	1.0	90
ADH1C*2	Gln272, Val350	γ_2_	0.6	40
ADH1C*352Thr	Thr 352^†^	—	—	—
*ADH4*		π	30	20
*ADH5*		χ	>1,000	100
*ADH6*		ADH6	?	?
*ADH7*		σ	30	1800

* The ^*^1, ^*^2, etc., designations represent particular alleles of the genes indicated.

† ADH1C*352Thr has been found in Native Americans as an additional variation on chromosomes with the Val350 characteristic of ADH1C*2 (Osier et al. 2002*a*); the protein has not been isolated for study.

NOTE: *K*_m_(ethanol) indicates the concentration of ethanol at which the enzyme works at 50 percent capacity. Turnover indicates how many molecules of ethanol the enzyme will convert to acetaldehyde in 1 minute at saturating ethanol concentrations.

SOURCE: Modified from Hurley et al. 2002.

**Table 3:** Aldehyde Dehydrogenase (ALDH) Genes and Proteins

Official Gene Name*	**Older Names**	**Chromosomal Location**	**Protein**	**Sequence^†^**
*ALDH1A1*	*ALDH1, RALDH1*	9q21.13	Cytosolic aldehyde dehydrogenase 1, ALDH1	NM_000690
*ALDH2*		12q24.2	Mitochondrial aldehyde dehydrogenase, ALDH2	NM_000689

* Gene symbol approved by the Human Genome Organization (HUGO) Gene Nomenclature Committee (www.gene.ucl.ac.uk/nomenclature/), as used by the National Center for Biotechnology Information (NCBI).

† Reference sequence number as listed in the NCBI RefSeq database (www.ncbi.nlm.nih.gov/RefSeq/).

## Footnotes

1 For a definition of this and other technical terms, see the glossary, p. 32.

2 In recent years, the nomenclature for these genes was revised and standardized by the Human Genome Organization (HUGO). Unfortunately, there is some confusion in the literature from some groups using nonstandard nomenclature. In this article, the standard nomenclature is used.

3 If both subunits are the same, the enzyme is a homodimer; if the two subunits differ, it is a heterodimer.

4 Note that current nomenclature numbers the amino acids for all proteins from the methionine that is found at the beginning of every initial translation product and which, in the vast majority of cases, is removed during posttranslational processing. Earlier nomenclature numbered the amino acids according to their position in the final, mature protein. Therefore, what is now called Arg48, for example, is denoted in earlier literature as Arg47.

5 Every person carries two copies of every gene, one inherited from the mother and one inherited from the father. These copies may or may not contain the same allele.

6 This substitution corresponds to position 487 in the mature ALDH2 enzyme.

## References

[b1-arh-30-1-5-13] Birley AJ, Whitfield JB, Neale MC (2005). Genetic time-series analysis identifies a major QTL for in vivo alcohol metabolism not predicted by in vitro studies of structural protein polymorphism at the ADH1B or ADH1C loci. Behavioral Genetics.

[b2-arh-30-1-5-13] Chen C-C, Lu R-B, Chen Y-C (1999a). Interaction between the functional polymorphisms of the alcohol-metabolism genes in protection against alcoholism. American Journal of Human Genetics.

[b3-arh-30-1-5-13] Chen YC, Lu RB, Peng GS (1999b). Alcohol metabolism and cardiovascular response in an alcoholic patient homozygous for the ALDH2*2 variant gene allele. Alcoholism: Clinical and Experimental Research.

[b4-arh-30-1-5-13] Chen HJ, Tian H, Edenberg HJ (2005). Natural haplotypes in the regulatory sequences affect human alcohol dehydrogenase 1C (ADH1C) gene expression. Human Mutation.

[b5-arh-30-1-5-13] Choi IG, Son HG, Yang BH (2005). Scanning of genetic effects of alcohol metabolism gene (ADH1B and ADH1C) polymorphisms on the risk of alcoholism. Human Mutation.

[b6-arh-30-1-5-13] Chou W-Y, Stewart MJ, Carr LG (1999). An A/G polymorphism in the promoter of mitochondrial aldehyde dehydrogenase (ALDH2): Effects of the sequence variant on transcription factor binding and promoter strength. Alcoholism: Clinical and Experimental Research.

[b7-arh-30-1-5-13] Crabb DW, Edenberg HJ, Bosron WF, Li T-K (1989). Genotypes for aldehyde dehydrogenase deficiency and alcohol sensitivity: The inactive ALDH2(2) allele is dominant. Journal of Clinical Investigation.

[b8-arh-30-1-5-13] Crabb DW, Matsumoto M, Chang D, You M (2004). Overview of the role of alcohol dehydrogenase and aldehyde dehydrogenase and their variants in the genesis of alcohol-related pathology. Proceedings of the Nutrition Society.

[b9-arh-30-1-5-13] Dickson PA, James MR, Heath AC (2006). Effects of variation at the ALDH2 locus on alcohol metabolism, sensitivity, consumption, and dependence in Europeans. Alcoholism: Clinical and Experimental Research.

[b10-arh-30-1-5-13] Duester G, Farres J, Felder MR (1999). Recommended nomenclature for the vertebrate alcohol dehydrogenase gene family. Biochemical Pharmacology.

[b11-arh-30-1-5-13] Edenberg HJ (2000). Regulation of the mammalian alcohol dehydrogenase genes. Progress in Nucleic Acid Research and Molecular Biology.

[b12-arh-30-1-5-13] Edenberg HJ, Foroud T (2006). The genetics of alcoholism: Identifying specific genes through family studies. Addiction Biology.

[b13-arh-30-1-5-13] Edenberg HJ, Jerome RE, Li M (1999). Polymorphism of the human alcohol dehydrogenase 4 (ADH4) promoter affects gene expression. Pharmacogenetics.

[b14-arh-30-1-5-13] Edenberg HJ, Xuei X, Chen HJ (2006). Association of alcohol dehydrogenase genes with alcohol dependence: A comprehensive analysis. Human Molecular Genetics.

[b15-arh-30-1-5-13] Ehlers CL, Spence JP, Wall TL (2004). Association of ALDH1 promoter polymorphisms with alcohol-related phenotypes in southwest California Indians. Alcoholism: Clinical and Experimental Research.

[b16-arh-30-1-5-13] Guindalini C, Scivoletto S, Ferreira RG (2005). Association of genetic variants in alcohol dehydrogenase 4 with alcohol dependence in Brazilian patients. American Journal of Psychiatry.

[b17-arh-30-1-5-13] Han Y, Oota H, Osier MV (2005). Considerable haplotype diversity within the 23kb encompassing the ADH7 gene. Alcoholism: Clinical and Experimental Research.

[b18-arh-30-1-5-13] Hansell NK, Pang D, Heath AC (2005). Erythrocyte aldehyde dehydrogenase activity: Lack of association with alcohol use and dependence or alcohol reactions in Australian twins. Alcohol and Alcoholism.

[b19-arh-30-1-5-13] Harada S, Okubo T, Nakamura T (1999). A novel polymorphism (–357G/A) of the ALDH2 gene: Linkage disequilibrium and an association with alcoholism. Alcoholism: Clinical and Experimental Research.

[b20-arh-30-1-5-13] Hasin D, Aharonovich E, Liu X (2002). Alcohol and ADH2 in Israel: Ashkenazis, Sephardics, and recent Russian immigrants. American Journal of Psychiatry.

[b21-arh-30-1-5-13] Higuchi S, Matsushita S, Imazeki H (1994). Aldehyde dehydrogenase genotypes in Japanese alcoholics. Lancet.

[b22-arh-30-1-5-13] Hurley TD, Edenberg HJ, Li T-K (2002). The pharmacogenomics of alcoholism. Pharmacogenomics: The Search for Individualized Therapies.

[b23-arh-30-1-5-13] Kitson KE (1999). Regulation of alcohol and aldehyde dehydrogenase activity: A metabolic balancing act with important social consequences. Alcoholism: Clinical and Experimental Research.

[b24-arh-30-1-5-13] Lee SL, Chau GY, Yao CT (2006). Functional assessment of human alcohol dehydrogenase family in ethanol metabolism: Significance of first-pass metabolism. Alcoholism: Clinical and Experimental Research.

[b25-arh-30-1-5-13] Luczak SE, Glatt SJ, Wall TJ (2006). Meta-analyses of ALDH2 and ADH1B with alcohol dependence in Asians. Psychological Bulletin.

[b26-arh-30-1-5-13] Luczak SE, Shea SH, Carr LG (2002). Binge drinking in Jewish and non-Jewish white college students. Alcoholism: Clinical and Experimental Research.

[b27-arh-30-1-5-13] Luo X, Kranzler HR, Zuo L (2005). ADH4 gene variation is associated with alcohol and drug dependence: Results from family controlled and population-structured association studies. Pharmacogenetics and Genomics.

[b28-arh-30-1-5-13] Luo X, Kranzler HR, Zuo L (2006). ADH4 gene variation is associated with alcohol dependence and drug dependence in European Americans: Results from HWD tests and case-control association studies. Neuropsychopharmacology.

[b29-arh-30-1-5-13] Mulligan CJ, Robin RW, Osier MV (2003). Allelic variation at alcohol metabolism genes (ADH1B, ADH1C, ALDH2) and alcohol dependence in an American Indian population. Human Genetics.

[b30-arh-30-1-5-13] O’Connor S, Morzorati S, Christian J, Li T-K (1998). Clamping breath alcohol concentration reduces experimental variance: Application to the study of acute tolerance to alcohol and alcohol elimination rate. Alcoholism: Clinical and Experimental Research.

[b31-arh-30-1-5-13] Oota H, Pakstis AJ, Bonne-Tamir B (2004). The evolution and population genetics of the ALDH2 locus: Random genetic drift, selection, and low levels of recombination. Annals of Human Genetics.

[b32-arh-30-1-5-13] Osier MV, Pakstis AJ, Kidd JR (1999). Linkage disequilibrium at the ADH2 and ADH3 loci and risk of alcoholism. American Journal of Human Genetics.

[b33-arh-30-1-5-13] Osier MV, Lu RB, Pakstis AJ (2004). Possible epistatic role of ADH7 in the protection against alcoholism. American Journal of Medical Genetics Part B Neuropsychiatric Genetics.

[b34-arh-30-1-5-13] Osier MV, Pakstis AJ, Goldman D (2002a). A proline-threonine substitution in codon 351 of ADH1C is common in Native Americans. Alcoholism: Clinical and Experimental Research.

[b35-arh-30-1-5-13] Osier MV, Pakstis AJ, Soodyall H (2002b). A global perspective on genetic variation at the ADH genes reveals unusual patterns of linkage disequilibrium and diversity. American Journal of Human Genetics.

[b36-arh-30-1-5-13] Peng GS, Wang MF, Chen CY (1999). Involvement of acetaldehyde for full protection against alcoholism by homozygosity of the variant allele of mitochondrial aldehyde dehydrogenase gene in Asians. Pharmacogenetics.

[b37-arh-30-1-5-13] Peng GS, Yin JH, Wang MF (2002). Alcohol sensitivity in Taiwanese men with different alcohol and aldehyde dehydrogenase genotypes. Journal of the Formosan Medical Association.

[b38-arh-30-1-5-13] Spence JP, Liang T, Eriksson CJ (2003). Evaluation of aldehyde dehydrogenase 1 promoter polymorphisms identified in human populations. Alcoholism: Clinical and Experimental Research.

[b39-arh-30-1-5-13] Thomasson HR, Edenberg HJ, Crabb DW (1991). Alcohol and aldehyde dehydrogenase genotypes and alcoholism in Chinese men. American Journal of Human Genetics.

[b40-arh-30-1-5-13] Wall TL, Carr LG, Ehlers CL (2003). Protective association of genetic variation in alcohol dehydrogenase with alcohol dependence in Native American Mission Indians. American Journal of Psychiatry.

[b41-arh-30-1-5-13] Warren KR, Li TK (2005). Genetic polymorphisms: Impact on the risk of fetal alcohol spectrum disorders. Birth Defects Research, Part A: Clinical and Molecular Teratology.

[b42-arh-30-1-5-13] Whitfield JB Alcohol dehydrogenase and alcohol dependence: Variation in genotype-associated risk between populations. American Journal of Human Genetics.

[b43-arh-30-1-5-13] Xiao Q, Weiner H, Crabb DW (1996). The mutation in the mitochondrial aldehyde dehydrogenase (ALDH2) gene responsible for alcohol-induced flushing increases turnover of the enzyme tetramers in a dominant fashion. Journal of Clinical Investigation.

[b44-arh-30-1-5-13] Xiao Q, Weiner H, Johnston T, Crabb DW (1995). The aldehyde dehydrogenase ALDH2*2 allele exhibits dominance over ALDH2*1 in transduced HeLa cells. Journal of Clinical Investigation.

